# Three-Component Reaction of Tautomeric Amidines with 3-Ferrocenylmethylidene-2,4-pentanedione. Formation of Polymeric Coordination Complexes of Potassium Ferrocenyl-(hexahydro)pyrimidoxides

**DOI:** 10.3390/molecules19010041

**Published:** 2013-12-20

**Authors:** Elena I. Klimova, Marcos Flores-Alamo, Tatiana Klimova, Sandra Cortez Maya, Irina P. Beletskaya

**Affiliations:** 1 Facultad de Química, Universidad Nacional Autónoma de México, Cd. Universitaria, Coyoacán, C.P. 04510, México D.F., Mexico; 2 Chemistry Department, Lomonosov Moscow State University, Leninskie Gory, Moscow119899, Russia

**Keywords:** ferrocene, 3-ferrocenylmethylidene-2,4-pentanedione, amidines, ferrocenyl-pyrimidines, ferrocenylpiperidones, complexes of potassium ferrocenyl(hexahydro)-pyramidin-4-oxides

## Abstract

Acetamidine hydrochloride and *p*-aminobenzamidine dihydrochloride interact with 3-ferrocenylmethylidene-2,4-pentanedione at 80–82 °C in the presence of K_2_CO_3_ in the water–alcohol medium in two tautomeric forms (the amidoimine and enediamine ones) with formation of mixtures of pyrimidine and piperidone derivatives and polymeric coordination complexes of potassium ferrocenyl(hexahydro)pyrimidoxides. The structure of the resultant compounds is elucidated on the basis of IR, ^1^H- and ^13^C-NMR spectroscopy, mass spectrometry and elemental analysis data. The crystal structures of 6-ferrocenyl-4-hydroxy-4-methyl-2-piperidone, potassium 6-ferrocenyl-4-methyl-2-methylidene(hexahydro)pyrimidin-4-oxide and 2-(4-aminophenyl)-4-ferrocenyl-6-methyl-pyrimidine were determined by X-ray analysis of suitable single crystals.

## 1. Introduction

Organic compounds of the ferrocene series evoke interest in view of their potential practical applications in diverse fields, such as organic and organometallic synthesis, coordination chemistry, materials science, homogeneous catalysis, supramolecular chemistry, chemo- and biosensors, medicinal chemistry, *etc.* [1,2,3,4,5,6,7]. Ferrocenyl-substituted nitrogen heterocycles are of special interest in the search for bioactive substances. Such compounds include ferrocenyl derivatives of pyrazolines, pyrazoles, triazoles, benzimidazoles, benzindazoles, quinuclidines, pyrimidines, *etc.* [8,9,10,11,12,13,14,15]. It is known that the presence of ferrocene groups in heterocyclic compounds usually enhances their pharmacological characteristics in comparison with substances of similar structure but without ferrocene substituents. Therefore, the keen interest in the synthesis of novel ferrocenyl-containing heterocycles is quite natural, especially if these compounds contain functional groups in heterocyclic rings. 

We have recently reported the synthesis of ethyl-2-aryl(methyl)- and 2-amino-6-ferroce-nyl(dihydro)pyrimidine-4-carboxylates with high anticarcinogenic activity [16] via the reaction between ethyl-2-acyl-3-ferrocenyl acrylates with amidines. Bioactive ferrocenyl(dihydro)pyrimidines with ethoxycarbonyl substituents in the heterocyclic nuclei were isolated with yields about 40%–45%, together with the products of several side processes: fragmentations of the initial ethyl 2-acyl-3-ferrocenyl acrylates, 1,3-insertion of amidines into ferrocenyl acrylates [16,17,18], *etc*. However, despite the comparatively low yields of ferrocenyl(dihydro)pyrimidines, the high bioactivity of the latter compounds evoked our interest in a more detailed investigation of the interaction between ferrocenyl-containing β-dicarbonyl compounds and nitrogen polynucleophiles.

## 2. Results and Discussion

### 2.1. Reactions of Acetamidine and p-Aminobenzamidine with 3-Ferrocenylmethylidene-2,4-pentanedione

**Scheme 1:**
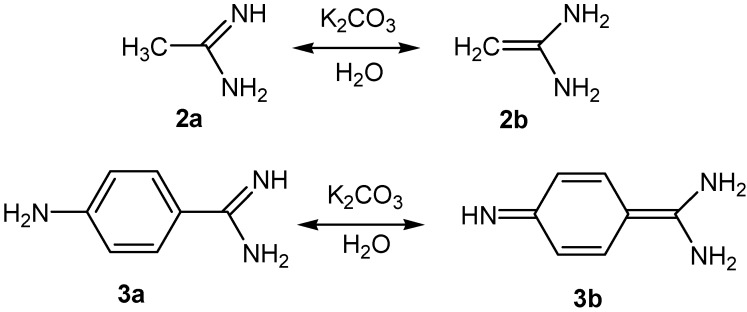
Tautomeric forms of the acetamidine (**2a**, **2b**) and *p*-aminobenzamidine (**3a**, **3b**).

This paper investigates the interaction of acetamidine hydrochloride (**2**) and *p*-aminobenzamidine dihydrochloride (**3**) with 3-ferrocenylmethylidene-2,4-pentanedione (**1**) in the presence of excess of K_2_CO_3_. Acetamidine and *p*-aminobenzamidine, which are formed from hydrochlorides **2** and **3** as a result of treatment with potassium carbonate in the water-alcohol medium used, must exist in two tautomeric forms: **2a** and **2b**, **3a** and **3b** (Scheme 1), respectively. Thus, 3-ferrocenylmethylidene-2,4-dione (**1**) must interact with each of the tautomeric forms **2a**, **2b** and **3a**, **3b** of the amidines.

**Scheme 2:**
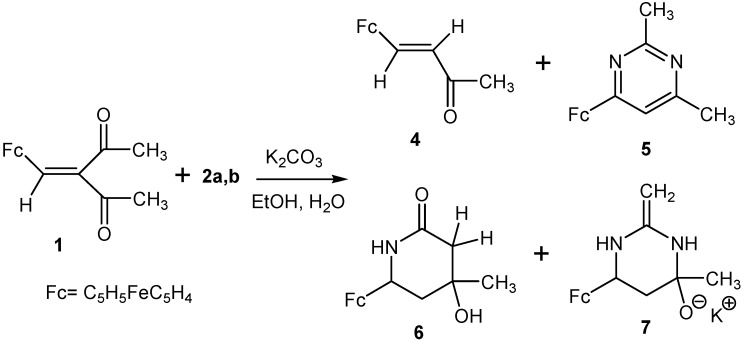
Reaction of 3-ferrocenylmethylidene-2,4-pentanedione (**1**) with acetamidine (**2a**,**b**).

Indeed, we have found that 1,3-diketone **1** reacts with acetamidines (**2a**,**b**) (water–alcohol medium, K_2_CO_3_, 80–85 °C), yielding a mixture of several products: 4-ferrocenyl-3-butenone (**4**, ~25%), 4-ferrocenyl-2,6-dimethylpyrimidine (**5**, ~20%), 6-ferrocenyl-4-hydroxy-4-methyl-2-piperidone (**6**, ~23%), and potassium 6-ferrocenyl-4-methyl-2-methylidene(hexahydro)pyrimidin-4-oxide (**7**, ~17%) (Scheme 2).

Compounds **4**–**7** were separated on a chromatographic column containing Al_2_O_3_ (Brockmann activity grade III). The first compound to leave the column is chalcone **4** (eluent: hexane–ether, 5:1), the next one is pyrimidine **5** (eluent: hexane–ether, 2:1), then hydroxypiperidone **6** (eluent: hexane–CH_2_Cl_2_, 2:1), and the last one to be washed out is potassium pyrimidoxide **7** (eluent: CH_2_Cl–CH_3_OH–H_2_O, 2:2:1).

The structure of compounds **4**–**7** was elucidated on the basis of elemental analysis, mass spectrometry, ^1^H and ^13^C-NMR spectroscopy data, as well as X-ray diffraction analysis of single crystals of compounds **6** and **7**.

According to ^1^H-NMR findings, 4-ferrocenyl-3-butenone (**4**) is generated solely in the form of the *trans*-isomer according to the *J* = 15.9 Hz spin-spin coupling constant of the olefin protons. The ^1^H-NMR spectrum of ferrocenylpyrimidine **5** contains signals from hydrogen atoms of two methyl groups and one ferrocene substituent, as well as one singlet from the olefinic hydrogen atom of the heterocycle. Additionally, in the ^13^C-NMR spectrum of compound **5**, there are three signals from the C_1_, C_3_ and C_5_ carbons of the heterocyclic ring and one signal from C_ipso_Fc, thus unambiguously confirming the structure of the resultant ferrocenylpyrimidine **5**.

The structure of hydroxypiperidone **6** was established on the basis of IR, ^1^H-NMR, and ^13^C-NMR spectroscopy. The IR spectrum of compound **6** contains characteristic absorption bands of the NH, OH, C=O, and Fc groups (see the Experimental section). The ^1^H-NMR spectrum contains signals from protons of one methyl and one ferrocene substituents, one hydroxyl group, one NH-fragment, two doublets from protons of the methylene fragment in the heterocycle, and also an ABX pattern of signals from protons of the –CH_2_–CH– fragment in the heterocycle. The ^13^C-NMR data also confirm the structure of compound **6**.

**Figure 1:**
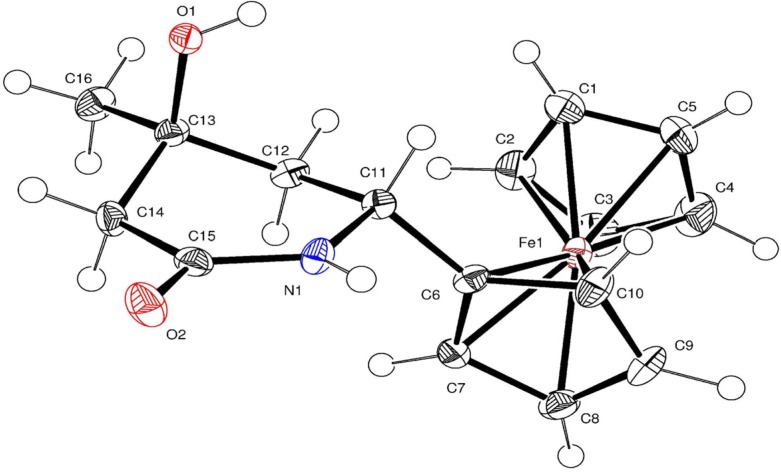
X-ray crystal structure of **6**.

In addition to spectral data, the spatial configuration of piperidone **6** was further confirmed by X-ray diffraction analysis of single crystals obtained by crystallization from CH_2_Cl_2_. The general view of molecule **6** is shown in Figure 1, while the principal geometric parameters are listed in the Table 1. The six-membered cycle in structure **6** has a boat conformation, where the ferrocene and methyl substituents occupy the axial-equatorial positions.

The structure of product **7**, isolated by chromatography with the use of water in the eluent, was determined according to the corresponding elemental analysis, mass spectrometry, IR, ^1^H-NMR, and ^13^C-NMR spectroscopy data. According to these findings, molecule **7** contains the following fragments: Me, Fc, CH_2_=, 2 NH and –CH_2_–CH–, in addition to two signals of quaternary carbons (δ = 55.01, 158.75) and one signal of C_ipsoFc_.

**Figure 2:**
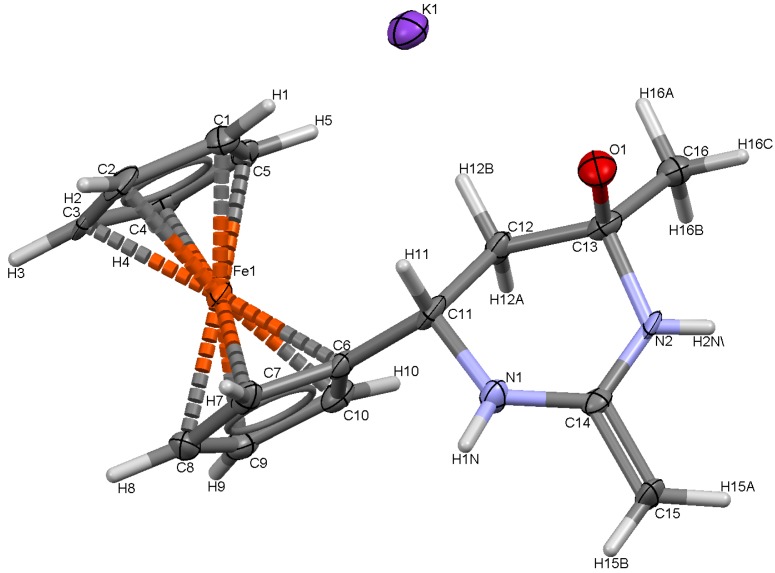
X-ray crystal structure of **7**.

Compound **7** is a water- and alcohol-soluble yellow crystalline substance, storage-stable in the crystalline form and in solutions, it decomposes during melting (~286 °C), a property that is characteristic of ionic products. Using crystallization from aqueous methanol, we have managed to grow single crystals of **7** that were suitable for X-ray diffraction studies of the spatial structure of **7**. The general view of molecule **7** is shown in Figure 2, and the main geometrical parameters are listed in Table 1.

**Table 1:** Selected bond lengths and bond angles for compounds **6**, **7** and **8**.

Selected bond lengths (Å)		Selected bond angles (°)	
**6**			
C(11)-N(1)	1.476(3)	N(1)-C(11)-C(12)	111.22(19)
C(11)-C(12)	1.520(3)	C(13)-C(12)-C(11)	113.77 (18)
C(12)-C(13)	1.517(3)	O(1)-C(13)-C(12)	111.54(19)
C(13)-O(1)	1.439(3),	O(1)-C(13)-C(14)	105.81(18)
C(15)-N(1)	1.329(3)	C(12)-C(13)-C(14)	108.05 (19)
C(13)-C(14)	1.524(3)	C(15)-C(14)-C(13)	112.94(19)
C(14)-C(15)	1.506(3)	O(2)-C(15)-N(1)	121.3(2)
C(15)-O(2)	1.247(3).	N(1)-C(15)-C(14)	117.9(2)
**7**			
N(1)-C(11)	1.482(6)	N(1)-C(11)-C(12)	109.6(4)
C(11)-C(12)	1.522(6)	C(13)-C(12)-C(11)	112.7(4)
C(12)-C(13)	1.520(7)	O(1)-C(13)-N(2)	105.1(4)
C(13)-O(1)	1.418(6)	O(1)-C(13)-C(12)	111.3(4)
C(13)-N(2)	1.469(6)	N(2)-C(13)-C(12)	106.8(4)
C(14)-N(2)	1.305(6)	N(2)-C(14)-N(1)	120.4(4)
C(14)-N(1)	1.327(6)	N(2)-C(14)-C(15)	120.5(4)
C(14)-C(15)	1.487(6)	N(1)-C(14)-C(15)	119.1(4)
O(1)-K(1)	3.072(4)	C(13)-O(1)-K(1)	119.5(3)
K(1)-N(1)#1	3.136(4)	O(1)-K(1)-N(1)#1	97.31(10)
N(1)-K(1)#1	3.136(4)	O(1)-K(1)-N(2)#2	138.86(11)
K(1)-N(2)#2	3.147(5)	N(1)#1-K(1)-N(2)#2	92.44(11)
N(2)-K(1)#3	3.147(5)	C(14)-N(2)-C(13)	123.1(4)
C(13)-C(16))	1.502(7)	C(14)- N(1)- K(1)#1	117.0(3)
C(1)-Fe(1)	2.044(5)	C(11)- N(1)- K(1)#1	116.9(3)
N(1)- H(N1)	0.91(5)	C(14)- N(2)- K(1)#3	121.2(3)
N(2)- H(N2)	0.76(5)	C(13)- N(2)- K(1)#3	115.4(3)
**8**			
N(1)-C(11)	1.347(5)	N(1)-C(11)-C(12)	121.4(3)
C(11)-C(12)	1.400(5)	C(13)-C(12)-C(11)	116.9(3)
C(12)-C(13)	1.389(6)	N(2)-C(13)-C(12)	121.8(3)
C(13)-N(2)	1.347(5)	N(2)-C(13)-C(21)	117.5(3)
C(14)-N(2)	1.340(5)	N(2)-C(14)-N(1)	125.3(3)
C(14)-N(1)	1.341(5)	N(2)-C(13)-C(15)	118.7(3)
C(14)-C(15)	1.470(5)	C(14)-N(1)-C(11)	117.3(3)
C(18)-N(3)	1.377(5)	C(14)-N(2)-C(13)	117.2(3)

**Figure 3:**
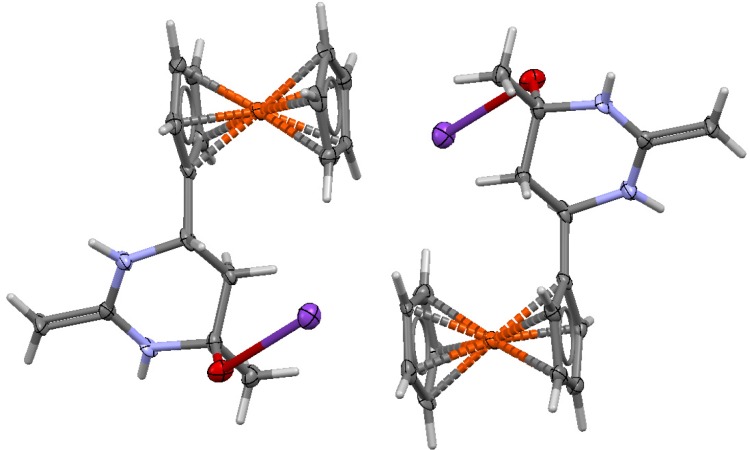
Structure of the fragment of unit cell of **7**.

As follows from the results of this analysis, compound **7** has the structure of potassium 6-ferrocenyl-4-methyl-2-methylidene(hexahydro)pyrimidin-4-oxide. The central fragment of the molecule is a six-membered ring with two nitrogen atoms. A characteristic feature of the crystal structure of **7** is that the unit cell contains two molecules with oppositely oriented ferrocene fragments and methylene fragments oriented so as to be brought closer to each other (Figure 3).

**Figure 4:**
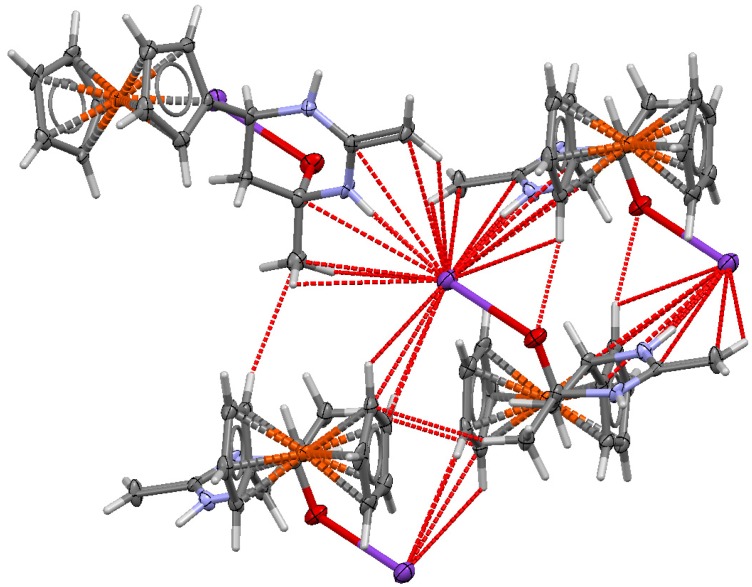
Fragment of X-ray crystal structure of **7**.

Figure 4 shows the nature of coordination interactions of the potassium cation with three adjacent molecules **7**, forming the polymeric structure of compound **7** and determining its high stability.

**Scheme 3:**
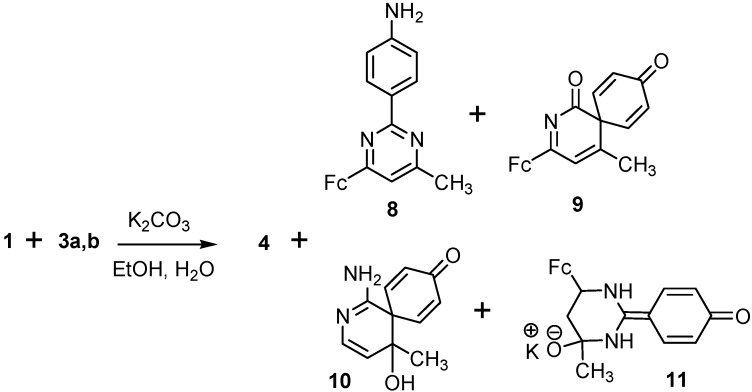
Reaction of 3-ferrocenylmethylidene-2,4-pentanedione (**1**) with 4-amino- benzamidine (**3a**,**b**).

The interaction of 3-ferrocenylmethylidene-2,4-pentanedione (**1**) with 4-aminobenzamidine (**3a**,**b**) dihydrochloride (EtOH/H_2_O, K_2_CO_3_, 80–82 °C) also leads to the formation of a mixture of several products: *trans*-4-ferrocenyl-3-butenone (**4**), 2-(4-aminophenyl)-4-ferrocenyl-6-methylpyrimidine (**8**), and three spiro compounds: **9**, **10**, and **11** (Scheme 3).

Compounds **4**, **8**–**11** were separated using column chromatography (Al_2_O_3_, grade III). The structure of each compound was determined on the basis of IR, ^1^H and ^13^C-NMR spectra, mass spectra, and elemental analysis, which are described in the Experimental section. 

**Figure 5:**
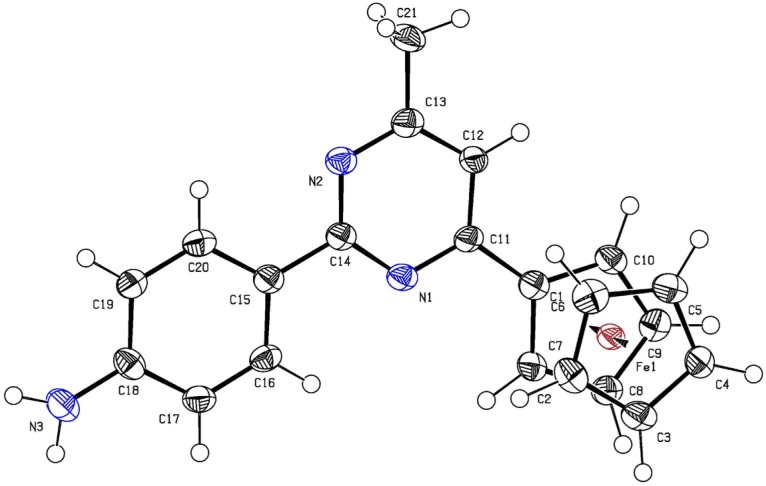
X-ray crystal structure of **8**.

X-ray analysis of single crystals of compound **8** obtained by crystallization from CH_2_Cl_2_ confirmed the structure of **8** to be that of 2-(4-aminophenyl)-4-ferrocenyl-6-methylpyrimidine (Figure 5). The X-ray analysis data show that the N-C bonds in pyrimidine **8** have virtually equal lengths [d = 1.347 (5), 1.340 (5) Å]. The lengths of Fe-C bonds and the geometry of the ferrocene sandwich are the same as in related compounds [19].

Compounds **9** and **10** are bright red powder substances, isolated in yields of about 10%–12%. The IR spectra of compounds **9** and **10** contain the corresponding numbers of characteristic C=O (compounds **9** and **10**), NH_2_ and OH (compound **10**), and Fc fragment absorption bands. The ^1^H-NMR spectra of compounds **9** and **10** contain signals from the unsubstituted cyclopentadienyl rings of ferrocene, together with signals from protons of substituted cyclopentadienyl rings, signals from methyl groups, from olefin protons (compounds **9** and **10**), from hydroxyl and amine groups (compound **10)**, and semiquinoid substituents. Each of the ^13^C-NMR spectra contains one signal from the quaternary spiro carbon atoms with δ = 48.31 ppm and 47.28 ppm, one signal of C_ipso_Fc, and the corresponding numbers of signals from carbon atoms of carbonyl groups and quaternary carbons of the heterocycles. The mass spectra of compounds **9** and **10** contain peaks of molecular ions with *m*/*z* = 371 and 378 [M]^+^, respectively; this fact also confirms the assumed structure.

Compound **11** is a yellow powder, which is soluble in water, methanol, and DMSO; it decomposes upon heating (~310 °C). The mass spectrum of compound **11** contains the peak of a molecular ion with *m*/*z* = 429 [M]^+^. The IR spectrum of compound **11** contains the characteristic absorption bands of the Fc, C=O, and NH groups. The ^1^H and ^13^C-NMR spectroscopy data are provided in the Experimental section. As follows from the ^1^H-NMR spectra, compound **11** was obtained in the form of one diastereomer, presumably with *a,e-trans*-oriented Fc and Me groups. On the basis of our data and by analogy with the structure of potassium pyrimidoxide **7**, product **11** was assumed to have the structure of potassium 6-ferrocenyl-4-methyl-2(4-oxo-2,5-cyclohexadienylidene)-hexahydropyrimid-4-oxide. Unfortunately, we failed to obtain single crystals of compound **11** that would be suitable for confirming its spatial structure by X-ray diffraction analysis. 

The results of this study (Scheme 2 and Scheme 3) show that all heterocyclic compounds **5**–**7** and **8**–**11** were formed via fragmentation of the initial β-diketone into chalcone **4**, whose cyclocondensation with amidines **2a**,**b** and **3a**,**b** yielded products **5**–**11**. In this process, pyrimidines **5** and **8** were formed from tautomers **2a** and **3a**, and compounds **6**, **7**, **9**–**11** were formed from tautomers **2b** and **3b**, respectively. 

**Scheme 4:**
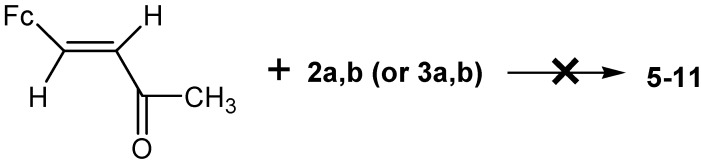
Reaction of 4-ferrocenyl-3-buten-2-one (**1**) with amidines (**2**) and (**3**).

To prove this statement, we studied the interaction of 4-ferrocenyl-3-buten-2-one **1** with amidines **2** and **3** under similar conditions (EtOH/H_2_O, K_2_CO_3_, 80–82 °C) and found that enone under these conditions does not cyclocondense with amidines **2** and **3** (Scheme 4).

The reaction almost quantitatively yielded the initial chalcone **4**, even after boiling of the reaction mixture for 24 h.

### 2.2. Posible Mechanisms of the Reactions of Acetamidine and p-Aminobenzamidine with 3-Ferrocenyl-methylidene-2,4-pentanedione

**Scheme 5:**
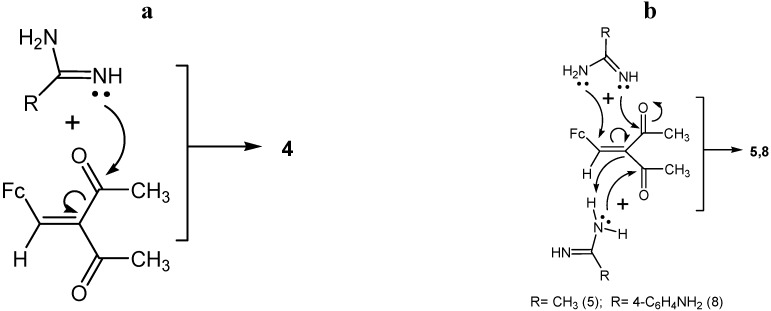
Plausible mechanisms for the formation of compound **4** (**a**), compounds **5** and **8** (**b**).

**Scheme 6:**
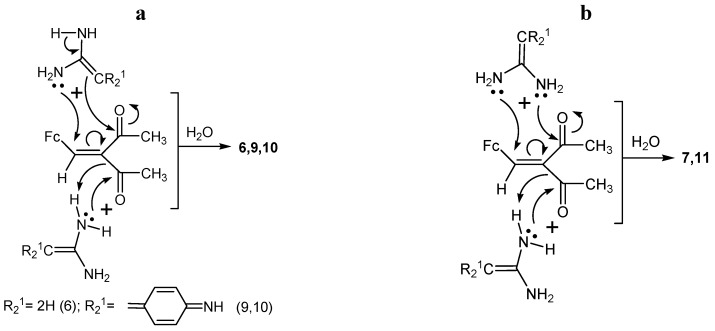
Plausible mechanisms for the formation of compounds **6**, **9**, **10** (**a**), compounds **7** and **11** (**b**).

On the basis of the results, we can make the following conclusions:

(1) Chalcone **4** is formed in the studied reactions as a result of one-pot nucleophilic attack of amidine nitrogens on the carbonyl carbon of the acetyl group in β-diketone **1** (Scheme 5a).
(2) Compounds **5**–**11** were formed in the three-component reaction with a simultaneous nucleophilic attack of two amidine molecules (**2** or **3**) on one β-diketone molecule. The presumable reaction mechanisms are shown in Scheme 5b and Scheme 6a,b.

## 3. Experimental

### 3.1. General

Column chromatography was carried out on alumina (Brockmann activity III). The ^1^H- and ^13^C-NMR spectra were recorded on a Unity Inova Varian spectrometer (300 and 75 MHz) for solutions in CDCl_3_ (for compounds **4**–**6** and **8**–**10**) and D_2_O (for compounds **7** and **11**), with Me_4_Si as the internal standard. The IR spectra were measured with a Perkin-Elmer Instruments Spectrum RXI FTIR spectrophotometer using KBr pellets. The mass spectra were obtained on a Varian MAT CH-6 instrument (EI MS, 70 eV). The melting pointa were determined with w micro melting point apparatus (Boëtius or Fisher) and the uncorrected values were used. An Elementar Analysensysteme LECO CHNS-900 apparatus was used for the elemental analyses. The following reagents were purchased from Aldrich (Toluca, Mexico): ferrocenecarbaldehyde, 99%; acetylacetone, 99+%; acetamidine hydrochloride, 95%; 4-aminobenzamidine dihydrochloride, 95%*.* 3-Ferrocenyl-methylidenepentane-2,4-dione (**1**) was prepared by condensation of ferrocenecarbaldehyde with acetylacetone in benzene in the presence of piperidinium acetate. The physical and ^1^H-NMR spectroscopic characteristics of compound **1** were all in accordance with the literature data [18,20,21].

### 3.2. Reactions of 3-Ferrocenylmethylidenepentane-2,4-dione (**1**) with Acetamidine Hydrochloride (**2**) (General Procedure)

A mixture of compound **1** (1.48 g, 5.0 mmol), acetamidine hydrochloride **(2**, 0.95 g, 10.0 mmol), ethanol (60 mL), H_2_O (10 mL) and K_2_CO_3_ (1.4 g) was stirred for 2 h at 80 °C. The solvents were removed *in vacuo*, the residue mixed with dichloromethane (50 mL) and Al_2_O_3_ (activity III) (20 g). The solvent was evaporated in air. This sorbent was applied onto a column with Al_2_O_3_ (the height of alumina is *ca*. 20 cm) and the reaction products were eluted from the column to afford the following reaction products: *trans-*4-ferrocenyl-3-buten-2-one (**4**, eluted with hexane–ether, 5:1), 4-ferrocenyl-2,6-dimethylpyrimidine (**5**, eluted with hexane–ether, 2:1), 6-ferrocenyl-4-hydroxy-4-methyl-2-piperidone (**6**, eluted with hexane–dichloromethane, 2:1), potassium 6-ferrocenyl-4-methyl-2-methyliden-hexahydro-4-pyrimidoxide (**7**, eluted with dichloromethane–methanol–water, 2:2:1).

*Trans-4-ferrocenyl-3-buten-2-one* (**4**). Red crystals, yield 0.32 g (25%, from **2**), 0.27 g (21 %, from **3**), m.p. 104–105 °C. ^1^H-NMR: 2.28 (s, 3H, CH_3_), 4.14 (s, 5H, C_5_H_5_), 4.14 (s, 5H, C_5_H_5_), 4.43 (m, 2H, C_5_H_4_), 4.50 (m, 2H, C_5_H_4_), 6.32 (d, 1H, CH=, *J* = 15.9 Hz), 7.42 (d, 1H, CH=, *J* = 15.9 Hz)

*4-Ferrocenyl-2,6-dimethylpyrimidine* (**5**). Orange crystals, yield 0.30 g (20%), m.p. 120–121 °C. IR (KBr): 491, 793, 820, 923, 1002, 1061, 1086, 1120, 1181, 1200, 1233, 1232, 1331, 1368, 1472, 1565, 1642, 1719, 2931, 2934, 2971, 3065, 3018, 3301 cm^−1^; ^1^H-NMR: 2.47 (s, 3H, CH_3_), 2.67 (s, 3H, CH_3_), 4.18 (s, 5H, C_5_H_5_), 4.45 (m, 2H, C_5_H_4_), 4.96 (m, 2H, C_5_H_4_), 7.01 (s, 1H, CH=); ^13^C-NMR: 29.18, 30.16 (2CH_3_), 69.58 (C_5_H_5_), 69.83, 71.12 (C_5_H_4_), 88.75 (C*_ipso_*Fc), 132.16 (CH=), 143.62, 152.19, 159.85 (3C). MS: *m/z* 292 [M]^+^. Anal. Calcd. for C_16_H_16_FeN_2_: C 65.78, H 5.52, Fe 19.12, N 9.58. Found: C 65.69, H 5.38, Fe 19.23, N 9.68.

*6-Ferrocenyl-4-hydroxy-4-methyl-2-piperidone* (**6**). Yellow crystals, yield 0.36 g (23%), m.p. 147–148 °C. IR (KBr): 512, 771, 805, 820, 953, 1002, 1045, 1079, 1103, 1147, 1198, 1223, 1309, 1348, 1376, 1489, 1571, 1668, 2956, 2980, 3324, 3429 cm^−1^; ^1^H-NMR: 1.36 (s, 3H, CH_3_), 1.51 (dd, 1H, CH_2_, *J* = 11.7, 13.2 Hz), 2.03 (dd, 1H, CH_2_, *J* = 4.17, 13.2 Hz), 2.12 (bs, 1H, OH), 2.39 (d, 1H, CH_2_, *J* = 17.7 Hz), 2.50 (d, 1H, CH_2_, *J* = 17.7 Hz), 4.22 (s, 5H, C_5_H_5_), 4.15 (m, 1H, C_5_H_4_), 4.17 (m, 2H, C_5_H_4_), 4.21 (m, 1H, C_5_H_4_), 4.54 (dd, 1H, CH, *J* = 4.16, 11.7 Hz), 6.18 (bs, 1H, NH); ^13^C-NMR: 30.14 (CH_3_), 44.70, 45.17 (2CH_2_), 64.63 (CH), 68.68 (C_5_H_5_), 66.68, 68.25, 68.39, 68.97 (C_5_H_4_), 90.54 (C*_ipso_*Fc), 49.07, 171.26 (2C); MS: *m/z* 313 [M]^+^. Anal. Calcd. for C_16_H_19_FeNO_2_: C 61.36, H 6.12, Fe 17.83, N 4.47. Found: C 61.18, H 6.16, Fe 17.79, N 4.58.

*Potassium 6-ferrocenyl-4-methyl-2-methyliden-4-hexahydropyrimidoxide* (**7**). Yellow crystals, yield 0.30 g (17%), m.p. dec.*ca* 302 °C. IR (KBr): 486, 502, 607, 771, 803, 821, 953, 1004, 1020, 1052, 1077, 1104, 1152, 1198, 1219, 1306, 1338, 1371, 1480, 1571, 1687, 2950, 2980, 3324 cm^−1^; ^1^H-NMR (D_2_O): 1.83 (s, 3H, CH_3_), 2.21 (t, 1H, CH_2_, *J* = 13.5 Hz), 2.43 (dd, 1H, CH_2_, *J* = 3.9, 13.5 Hz), 4.54 (dd, 1H, CH, *J* = 3.9, 13.5 Hz), 4.25 (s, 5H, C_5_H_5_), 4.28 (m, 1H, C_5_H_4_), 4.31 (m, 1H, C_5_H_4_), 4.34 (m, 1H, C_5_H_4_), 4.42 (m, 1H, C_5_H_4_), 4.88 (bs, 1H, NH), 4.93 (bs, 1H, NH), 5.17 (d, 1H, CH_2_=, *J* = 1.2 Hz), 5.31 (d, 1H, CH_2_=, *J* = 1.2 Hz); ^13^C-NMR (D_2_O): 18.19 (CH_3_), 38.89, 112.34 (2CH_2_), 46.63 (CH), 68.75 (C_5_H_5_), 65.91, 68.04, 68.15, 69.04 (C_5_H_4_), 76.87 (C*_ipso_*Fc), 55.01, 158.75 (2C); MS: *m/z* 350 [M]^+^. Anal. Calcd. for C_16_H_19_FeKN_2_O: C 54.88, H 5.47, N 7.99. Found: C 55.01, H 5.36, N 8.05.%.

### 3.3. Reactions of 3-Ferrocenylmethylidene-pentane-2,4-dione (**1**) with p-Aminobenzamidine Dihydrochloride (**3**)

Following the general procedure, the reaction of **1** (1.48 g, 5.0 mmol) with *p*-aminobenzamidine dihydrochloride (**3**, 2.07 g, 10.0 mmol) in a mixture of ethanol (60 mL) and H_2_O (10 mL) in the presence of K_2_CO_3_ (3.0 g) for 2 h at 80 °C afforded compounds **4** and **8**–**11**.

*2-(4-Aminophenyl)-4-ferrocenyl-6-methylpyrimidine* (**8**). Orange crystals, yield 0.44 g (24%), m.p. 153–154 °C. IR (KBr): 483, 545, 593, 771, 824, 840, 1001, 1023, 1104, 1176, 1254, 1303, 1325, 1376, 1438, 1489, 1522, 1570, 1585, 1621, 1725, 2854, 2924, 3082, 3096, 3210, 3364, 3459 cm^−1^; ^1^H-NMR: 2.53 (s, 3H, CH_3_), 3.90 (bs, 2H, NH_2_), 4.07 (s, 5H, C_5_H_5_), 4.47 (m, 2H, C_5_H_4_), 5.05 (m, 2H, C_5_H_4_), 6.77 (d, 2H, C_6_H_4_, *J* = 8.7 Hz), 6. 97 (s, 1H, CH=), 8.36 (d, 2H, C_6_H_4_, *J* = 8.7 Hz); ^13^C-NMR: 24.56 (CH_3_), 70.01 (C_5_H_5_), 68.09, 70.77 (C_5_H_4_), 81.52 (C*_ipso_*Fc), 112.69 (CH=), 114.72, 129.88 (C_6_H_4_), 128.87, 148.70, 163.99, 165.97, 167.16 (5C); MS: *m/z* 369 [M]^+^. Anal. Calcd. for C_21_H_19_FeN_3_: C 68.31, H 5.19, N 11.38. Found: C 68.32, H 5.17, N 11.26.

*3-Ferrocenyl-5-methyl-2-azaspiro[5.5]undeca-2,4,7,10-tetraen-1,9-dione* (**9**). Orange crystals, yield 0.22 g (12%), 176–178 °C. IR (KBr): 498, 681, 812, 835, 1002, 1026, 1039, 1105, 1196, 1206, 1226, 1280, 1301, 1344, 1394, 1441, 1469, 1519, 1547, 1575, 1599, 1622, 1698, 1705, 2949, 3197, 3210, 3345 cm^−1^; ^1^H-NMR: 2.13 (s, 3H, CH_3_), 4.21 (s, 5H, C_5_H_5_), 4.25 (m, 2H, C_5_H_4_), 4.39 (m, 2H, C_5_H_4_), 6.71 (d, 2H, C_6_H_4_, *J* = 8.7 Hz), 7. 41 (s, 1H, CH=), 7.52 (d, 2H, C_6_H_4_, *J* = 8.7 Hz); ^13^C-NMR: 29.64 (CH_3_), 69.18 (C_5_H_5_), 68.87, 69.96 (C_5_H_4_), 89.71 (C*_ipso_*Fc), 113.11 (CH=), 121.42, 131.33 (C_6_H_4_), 48.31, 58.45, 132.21 , 154.34, 154.89 (5C); MS: *m/z* 371 [M]^+^. Anal. Calcd. for C_21_H_17_FeNO_2_: C 67.94, H 4.62, N 3.77. Found: C 68.03, H 4.75, N 3.69.

*1-Amino-3-ferrocenyl-5-hydroxy-5-methyl-2-azaspiro[5.5]undeca-1,3,7,10-tetraen-9-one* (**10**). Orange crystals, yield 0.25 g (13%), m.p. 181–182 °C. IR (KBr): 486, 559, 725, 822, 842, 998, 1001, 1043, 1109, 1197, 1231, 1256, 1273, 1328, 1379, 1445, 1520, 1557, 1595, 1608, 1646, 1684, 1725, 2967, 3091, 3208, 3352, 3461 cm^−1^; ^1^H-NMR: 1.98 (s, 3H, CH_3_), 2.48 (bs, 1H, OH), 4.23 (s, 5H, C_5_H_5_), 4.17 (m, 2H, C_5_H_4_), 4.27 (m, 2H, C_5_H_4_), 5.03 – 5.23 (bs, 2H, NH_2_), 6.22 (s, 1H, CH=), 6.65 (d, 2H, C_6_H_4_, *J* = 7.8 Hz), 7.55 (d, 2H, C_6_H_4_, *J* = 7.8 Hz); ^13^C-NMR: 18.96 (CH_3_), 68.58 (C_5_H_5_), 66.02, 66.77, 67.90, 68.21 (C_5_H_4_), 90.93 (C*_ipso_*Fc), 103.89 (CH=), 112.95, 130.21 (C_6_H_4_), 47.28, 59.01, 130.19 , 153.11, 153.56 (5C); MS: *m/z* 388 [M]^+^. Anal. Calcd. for C_21_H_20_FeN_2_O_2_: C 64.97, H 5.19, N 7.21. Found: C 64.84, H 5.23, N 7.30.

*Potassium 6-ferrocenyl-4-methyl-2-(4-oxo-2,5-cyclohexadienyliden)-4-hexahydropyrimidoxide* (**11**). Yellow powder, yield 0.39 g (18%), m.p. dec.*ca* 298 °C. IR (KBr): 486, 553, 612, 715, 819, 913, 1000, 1026, 1078, 1102, 1201, 1272, 1337, 1421, 1432, 1478, 1557, 1631, 1689, 1714, 2861, 2929, 3046, 3323 cm^−1^; ^1^H-NMR: 1.85 (s, 3H, CH_3_), 2.09 (t, 1H, CH_2_, *J* = 13.2 Hz), 2.56 (dd, 1H, CH_2_, *J* = 4.2, 13.2 Hz), 4.83 (dd, 1H, CH, *J* = 4.2, 13.2 Hz), 4.23 (s, 5H, C_5_H_5_), 4.24 (m, 2H, C_5_H_4_), 4.45 (m, 2H, C_5_H_4_), 5.98 - 6.05 (bs, 2H, 2NH), 6.64 (d, 2H, C_6_H_4_, *J* = 8.7 Hz), 7.39 (d, 2H, C_6_H_4_, *J* = 8.7 Hz); ^13^C-NMR: 19.01 (CH_3_), 46.17 (CH_2_), 50.07 (CH), 68.68 (C_5_H_5_), 66.05, 67.85, 68.03, 69.40 (C_5_H_4_), 85.13 (C*_ipso_*Fc), 112.62, 130.11 (C_6_H_4_), 55.03, 130.88, 142.12, 167.51 (4C); MS: *m/z* 388 [M]^+^. Anal. Calcd. for C_21_H_21_FeKN_2_O_2_: C 58.76, H4.93, N 6.53. Found: C 58.87, H 5.01, N 6.42.

### 3.4. Crystal Structures of **6**, **7** and **8**

Single crystals of **6** and **8** were obtained by crystallization from CH_2_Cl_2_, while crystals of **7** were obtained by crystallization from H_2_O. The unit cell parameters and the X-ray diffraction intensities of **6**, **7** and **8** were recorded on a Gemini (detector Atlas CCD, Cryojet N_2_) diffractometer. The crystallographic data, the parameters of the X-ray diffraction experiments, and refinements are listed in supplementary Table S2. The structure of compounds **6**–**8** were solved by the direct method (SHELXS-97 [22]) and refined using full-matrix least-squares on *F*^2^.

*Crystal data for C_16_H_19_FeNO_2_* (**6**): M = 313.17 g·mol^−1^, orthorhombic P b c a, *a* = 10.5206(6), *b* = 10.1711(5), *c* = 25.8276(12) Å, α = 90, β = 90, γ = 90°, V = 2763.7(2) Å^3^, T = 130(2) K, Z = 8, ρ = 1.505 Mg/m^3^, wavelength 0.71073 Å, F(000) = 1312, absorption coefficient 1.091 mm^−1^, scan range 3.66 ≤ *θ* ≤ 26.05°, 2713 independent reflections, R_int_ = 0.0663, 20243 total reflections, 188 refinable parameters, final R indices [I > 2σ(I)] R_1_ = 0.0343, wR_2_ = 0.0677, R indices (all data) R_1_ = 0.0558, wR_2_ = 0.0763, goodness-of-fit on F^2^ 1.087, largest difference peak and hole 0.333/−0.318 eÅ^−3^.

*Crystal data for C_32_H_38_Fe_2_K_2_N_4_O_2_* (**7**): M = 700.56 g·mol^−1^, monoclinic P21/c, *a* = 10.5530(5), *b* = 9.8630(5), *c* = 15.1210(7) Å, α = 90, β = 101.702(5), γ = 90°, V = 1541.15(13) Å^3^, T = 130(2) K, Z = 2, ρ = 1.510 Mg/m^3^, wavelength 0.71073 Å, F(000) = 728, absorption coefficient 1.248 mm^−1^, scan range 3.34 ≤ *θ* ≤ 26.13°, 3041 independent reflections, R_int_ = 0.0830, 11105 total reflections, 199 refinable parameters, final R indices [I > 2σ(I)] R_1_ = 0.0555, wR_2_ = 0.1266, R indices (all data) R_1_ = 0.0993, wR_2_ = 0.1357, goodness-of-fit on F^2^ 1.008, largest difference peak and hole 0.814/−0.789 eÅ^−3^.

*Crystal data for C_21_H_19_FeN_3_* (**8**): M = 369.24 g·mol^−1^, monoclinic P21/c, *a* = 11.0190(5), *b* = 14.3412(5), *c* = 12.1698(4) Å, α = 90, β = 101.661(4), γ = 90°, V = 1883.45(12) Å^3^, T = 293(2) K, Z = 4, ρ = 1.302 Mg/m^3^, wavelength 1.54180 Å, F(000) = 768, absorption coefficient 6.460 mm^−1^, scan range 4.10 ≤ *θ* ≤ 66.59°, 3317 independent reflections, R_int_ = 0.0839, 11651 total reflections, 233 refinable parameters, final R indices [I > 2σ(I)] R_1_ = 0.0431, wR_2_ = 0.1652, R indices (all data) R_1_ = 0.0768, wR_2_ = 0.1779, goodness-of-fit on F^2^ 0.979, largest difference peak and hole 1.419/−0.539 eÅ^−3^.

CCDC-927702 (for **6**), CCDC-927704 (for **7**) and CCDC-927703 (for **8**) contain the supplementary crystallographic data for this paper. This data can be obtained free of charge at www.ccdc.cam.ac.uk/const/retrieving.html [or from the Cambridge Crystallographic Data Centre, 12, Union Road, Cambridge DB2 1EZ, UK; Fax: (internat.) +44 1223/336 033; E-mail: deposit@ccdc.cam.ac.uk].

## 4. Conclusions

Thus, it has been found that the two tautomeric forms (amidoimine and ene-1,1-diamine) of acetamidine (**2**) and *p*-aminobenzamidine (**3**) react with 3-ferrocenylmethylidene-2,4-pentanedione (**1**). Such an observation has been made for the first time. Two processes take place during the reaction: fragmentation of β-diketone **1** into β-ferrocenylvinyl(methyl)ketone **4** and three-component cyclocondensation–fragmentation, which leads to simultaneous formation of mixtures of ferrocenylpyrimidine and piperidone derivatives **5**–**11**, including spiro compounds (**9**, **10**), and polymeric coordination compounds: potassium hexahydropyrimidoxides (**7**, **11**). The results are so unexpected that they surely deserve more detailed investigation using a broader spectrum of reagents, in order to have more opportunities for practical studies of such processes. Synthesis of stable polymeric complexes of alkaline metals and studies of their physicochemical, biological, *etc.* properties are of special interest. 

## Supplementary Materials

Supplementary materials can be accessed at: http://www.mdpi.com/1420-3049/19/01/0041/s1.
