# Supplementary material for: Three-Component Reaction of Tautomeric Amidines with 3-Ferrocenylmethylidene-2,4-pentanedione. Formation of Polymeric Coordination Complexes of Potassium Ferrocenyl-(hexahydro)pyrimidoxides
Source: Molecules. 2013 Dec 20;19(1):41–54. doi: 10.3390/molecules19010041 (PMC6271121; doi:10.3390/molecules19010041)
Supplement: Supplementary file 1 [file molecules-19-00041-s001.pdf]

Supplementary materials

Table S1. Selected bond lengths and angles for compounds **7**, **6** and **8**.

**Bond lengths [Å]**		**Bond angles [°]**	
		**7**	
N(1)-C(11)	1.482(6)	N(1)-C(11)-C(12)	109.6(4)
C(11)-C(12)	1.522(6)	C(13)-C(12)-C(11)	112.7(4)
C(12)-C(13)	1.520(7)	O(1)-C(13)-N(2)	105.1(4)
C(13)-O(1)	1.418(6)	O(1)-C(13)-C(12)	111.3(4)
C(13)-N(2)	1.469(6)	N(2)-C(13)-C(12)	106.8(4)
C(14)-N(2)	1.305(6)	N(2)-C(14)-N(1)	120.4(4)
C(14)-N(1)	1.327(6)	N(2)-C(14)-C(15)	120.5(4)
C(14)-C(15)	1.487(6)	N(1)-C(14)-C(15)	119.1(4)
O(1)-K(1)	3.072(4)	C(13)-O(1)-K(1)	119.5(3)
K(1)-N(1)#1	3.136(4)	O(1)-K(1)-N(1)#1	97.31(10)
N(1)-K(1)#1	3.136(4)	O(1)-K(1)-N(2)#2	138.86(11)
K(1)-N(2)#2	3.147(5)	N(1)#1-K(1)-N(2)#2	92.44(11)
N(2)-K(1)#3	3.147(5)	C(14)-N(2)-C(13)	123.1(4)
C(13)-C(16))	1.502(7)	C(14)-N(1)-K(1)#1	117.0(3)
C(1)-Fe(1)	2.044(5)	C(11)-N(1)-K(1)#1	116.9(3)
N(1)-H(N1)	0.91(5)	C(14)-N(2)-K(1)#3	121.2(3)
N(2)-H(N2)	0.76(5)	C(13)-N(2)-K(1)#3	115.4(3)
		**6**	
C(11)-N(1)	1.476(3)	N(1)-C(11)-C(12)	111.22(19)
C(11)-C(12)	1.520(3)	C(13)-C(12)-C(11)	113.77(18)
C(12)-C(13)	1.517(3)	O(1)-C(13)-C(12)	111.54(19)
C(13)-O(1)	1.439(3)	O(1)-C(13)-C(14)	105.81(18)
C(13)-C(16)	1.518(3)	C(12)-C(13)-C(14)	108.05(19)
C(13)-C(14)	1.524(3)	C(15)-C(14)-C(13)	112.94(19)
C(15)-O(2)	1.247(3)	O(2)-C(15)-N(1)	121.3(2)
C(14)-C(15)	1.506(3)	O(2)-C(15)-C(14)	120.7(2)
C(15)-N(1)	1.329(3)	N(1)-C(15)-C(14)	117.9(2)
		**8**	
N(1)-C(11)	1.347(5)	N(1)-C(11)-C(12)	121.4(3)
C(11)-C(12)	1.400(5)	C(13)-C(12)-C(11)	116.9(3)
C(12)-C(13)	1.389(6)	N(2)-C(13)-C(12)	121.8(3)
C(13)-N(2)	1.347(5)	N(2)-C(13)-C(21)	117.5(3)
C(14)-N(2)	1.340(5)	N(2)-C(14)-N(1)	125.3(3)
C(14)-N(1)	1.341(5)	N(2)-C(13)-C(15)	118.7(3)
C(14)-C(15)	1.470(5)	C(14)-N(1)-C(11)	117.3(3)
C(18)-N(3)	1.377(5)	C(14)-N(2)-C(13)	117.2(3)
C(13)-C(16)	1.518(3)	C(15)-N(1)-C(11)	128.0 (2)

Table S2. Crystal data and structure refinement parameters for compounds **6**, **7** and **8**.

Data	6	7	8
Molecular formula	$C_{16}H_{19}Fe NO_2$	$C_{32}H_{38}Fe_2 K_2 N_4O_2$	$C_{21}H_{19}FeN_3$
Formula weight (g mol^{-1})	313.17	700.56	369.24
Temperature (K)	130(2)	130(2)	130 (2)
Crystal system	Orthorhombic	Monoclinic	Monoclinic
Space group	P b c a	P21/c	P21/c
a (Å)	10.5206(6)	10.5530(5)	11.0190(5)
b(Å)	10.1711(5)	9.8630(5)	14.3412(5)
c (Å)	25.8276(12)	15.1210(7)	12.1698(4)
α (°)	90	90	90
β (°)	90	101.702(5)	101.661(4)
γ (°)	90	90	90
V (Å^3)	2763.7(2)	1541.15(13)	1883.45(12)
Z	8	2	4
D calc. (mg/m^3)	1.505	1.510	1.302
Wavelength (Å)	0.71073	0.71073	1.54180
Absorption coefficient (mm^{-1})	1.091	1.248	6.460
F(000)	1312	728	768
θ range (°)	3.66–26.05	3.34–26.13	4.10–66.59
Reflections collected	20242	11105	11651
Reflections independent	2713	3041	3317
R_{int}	0.0663	0.0830	0.0839
Final R indices [I > 2σ(I)]	R_1 = 0.0343	R_1 = 0.0555	R_1 = 0.0431
	wR_2 = 0.0677	wR_2 = 0.1266	wR_2 = 0.1652
R indices (all data)	R_1 = 0.0558	R_1 = 0.0993	R_1 = 0.0768
	wR_2 = 0.0763	wR_2 = 0.1357	wR_2 = 0.1779
Refinable parameters	188	199	233
Goodness-of-fit on F^2	1.087	1.008	0.979
Maximum/minimum residual electron density (eÅ^{-3})	0.333/−0.318	0.814/−0.789	1.419/−0.539

Table S3. IR spectral data of compounds **6**, **7**, **8**, **9**, **10** and **11.**

No.	ν $_{max}$ (KBr)/cm^{-1}
**6**	512, 771, 805, 820, 953, 1002, 1045, 1079, 1103, 1147, 1198, 1223, 1309, 1348, 1376, 1489, 1571, 1668, 2956, 2980, 3324, 3429.
**7**	486, 502, 607, 771, 803, 821, 953, 1004, 1020, 1052, 1077, 1104, 1152, 1198, 1219, 1306, 1338, 1371, 1480, 1434, 1571, 1687, 2950, 2980, 3324.
**8**	483, 545, 593, 771, 824, 840, 1001, 1023, 1104, 1176, 1254, 1303, 1325, 1376, 1438, 1489, 1522, 1570, 1585, 1621, 1725, 2854, 2924, 3082, 3096, 3210, 3364, 3459.
**9**	498, 681, 812, 835, 1002, 1026, 1039, 1105, 1196, 1206, 1226, 1280, 1301, 1344, 1394, 1441, 1469, 1519, 1547, 1575, 1599, 1622, 1698, 1705, 2949, 3197, 3210, 3345.
**10**	486, 559, 725, 822, 842, 998, 1001, 1043, 1109, 1197, 1231, 1256, 1273, 1328, 1379, 1445, 1520, 1557, 1595, 1608, 1646, 1684, 1725, 2967, 3091, 3208, 3352, 3461.
**11**	486, 553, 612, 715, 819, 913, 1000, 1026, 1078, 1102, 1201, 1272, 1337, 1421, 1432, 1478, 1557, 1631, 1689, 1714, 2861, 2929, 3046, 3323.

Table S4. ^{1}H-NMR spectral data of compounds **5–11** (δ/ppm, *J*/Hz).

No.	C_5H_5 (s), C_6H_4 (m)	C_5H_4 (m)	CH_2 (m), CH=, OH (bs)	CH_3 (s),NH (bs), NH_2 (bs)
**5**	4.18 (5H)	4.45 (2H), 4.96 (2H)	7.01 (s, 1H)	2.47, 2.67
**6**	4.22 (5H)	4.43 (4H), 4.94 (4H)	1.51 (dd, 1H, *J* = 11.7, 13.2), 2.03 (dd, 1H, *J* = 4.17, 13.2), 2.12 (bs, 1H, OH), 2.39 (d, 1H, *J* = 17,7), 2.50 (d, 1H, *J* =17,7)	1.36
**7**	4.25 (5H)	4.28 (1H), 4.31(1H), 4.34 (1H), 4.42 (1H)	2.21 (t, 1H, *J* = 13.5), 2.43 (dd, 1H, *J* = 3.9, 13.5), 4.54 (dd, 1H, *J* = 3.9, 13.5), 5.17 (d, 1H, *J* = 1.2), 5.31 (d, 1H, *J* = 1.2)	1.83 (s), 4.88 (bs), 4.93 (bs)
**8**	4.07 (5H), 6.77 (2H), 8.36 (2H)	4.47 (2H), 5.05 (2H)	6.97 (s, 1H)	2.53 (s), 3.90 (bs, 2H, NH_2)
**9**	4.21(5H), 6.71 (2H), 7.52 (2H)	4.25 (2H), 4.39 (2H)	7.41 (s, 1H)	2.13 (s)
**10**	4.23 (5H), 6.65 (2H), 7.55 (2H)	4.17 (2H), 4.27 (2H)	6.22 (s, 1H)	1.98 (s), 2.48 (bs, OH). 5.03–5.23 (bs, 2H, NH_2)
**11**	4.23 (5H), 6.64 (2H), 7.39 (2H)	4.24 (2H), 4.45 (2H)	2.09 (t, 1H, *J* = 13.2), 2.56 (dd, 1H, *J* = 4.2, 13.2), 4.83 (dd, 1H, *J* = 4.2, 13.2),	1.85 (s), 5.98–6.05 (bs, 2H, 2NH)

Table S5. ^{13}C-NMR spectral data of compounds **5–11** (δ, ppm).

No.	C_5H_5, C_6H_4	C_5H_4	$C_{ipso}Fc$	CH_3	CH_2, CH, CH=	CH_2=, C
**5**	69.58	69.83, 71.12	88.75	29.18, 30.16	132.16	143.62, 152.19, 159.85
**6**	68.68	66.68, 68.25, 68.39, 68.97	90.54	30.14	44.70, 45.17, 64.63	49.07, 171.26
**7**	68.75	65.91, 68.04, 68.15, 69.04	76.87	18.19	38.89, 46.63,	112.34, 55.01, 158.75
**8**	70.01, 114.72, 129.88	68.09, 70.77	81.52	24.56	112.69	128.87, 148.70, 163.99, 165.97, 167.16
**9**	69.18, 121.42, 131.33	68.87, 69.96	89.71	29.64	113.11	48.31, 58.45, 132.21, 154.34, 154.89
**10**	68,58, 112.95, 130.21	66.02, 66.77, 67.90, 68.21	90.93	18.96	103.89	47.28, 59.01, 130.19, 153.11, 153.56
**11**	68.68, 112.62, 130.11	66.05, 67.85, 68.03, 69.40	85.13	19.01	46.17, 50.07	55.03, 130.88, 142.12, 167.51
